# Supplementary material for: HIVprotI: an integrated web based platform for prediction and design of HIV proteins inhibitors
Source: J Cheminform. 2018 Mar 9;10:12. doi: 10.1186/s13321-018-0266-y (PMC5845081; doi:10.1186/s13321-018-0266-y)
Supplement: Supplementary file 2 — Additional file 2. Source code of HIVProtI web server. [file 13321_2018_266_MOESM2_ESM.zip › HIVprotI_Source-code/hivprotI/weka-3-6-6/doc/weka/core/matrix/Maths.html]

Maths


---


|  |  |  |
| --- | --- | --- |
| **Overview** \| **Package** \| **Class** \| **Tree** \| **Deprecated** \| **Index** \| **Help** \| **Weka's home** | |  |
| **PREV CLASS**   **NEXT CLASS** | **FRAMES**    **NO FRAMES**     **All Classes** |  |
| SUMMARY: NESTED \| FIELD \| CONSTR \| METHOD | DETAIL: FIELD \| CONSTR \| METHOD |  |


---


## weka.core.matrix Class Maths

```
java.lang.Object
  weka.core.matrix.Maths
```

**All Implemented Interfaces:**: RevisionHandler

---

``` public class Maths extends java.lang.Object implements RevisionHandler ```

Utility class.

Adapted from the JAMA package.

**Version:**
:   $Revision: 1.3 $

**Author:**
:   The Mathworks and NIST, Fracpete (fracpete at waikato dot ac dot nz)

---

| **Field Summary** | |
| --- | --- |
| `static int` | `chisqDistribution`             Distribution type: chi-squared |
| `static double` | `logPSI`             The constant - log( sqrt(2 pi) ) |
| `static int` | `normalDistribution`             Distribution type: noraml |
| `static double` | `PSI`             The constant 1 / sqrt(2 pi) |
| `static int` | `undefinedDistribution`             Distribution type: undefined |


**Constructor Summary**

`Maths()`


| **Method Summary** | |
| --- | --- |
| `static double` | `dchisq(double x)`             Returns the density of the Chi-squared distribution. |
| `static double` | `dchisq(double x, double ncp)`             Returns the density of the noncentral Chi-squared distribution. |
| `static DoubleVector` | `dchisq(double x, DoubleVector ncp)`             Returns the density of the noncentral Chi-squared distribution. |
| `static double` | `dchisqLog(double x)`             Returns the log-density of the noncentral Chi-square distribution. |
| `static double` | `dchisqLog(double x, double ncp)`             Returns the log-density value of a noncentral Chi-square distribution. |
| `static DoubleVector` | `dchisqLog(double x, DoubleVector ncp)`             Returns the log-density of a set of noncentral Chi-squared distributions. |
| `static double` | `dnorm(double x)`             Returns the density of the standard normal. |
| `static double` | `dnorm(double x, double mean, double sd)`             Returns the density value of a standard normal. |
| `static DoubleVector` | `dnorm(double x, DoubleVector mean, double sd)`             Returns the density values of a set of normal distributions with different means. |
| `static double` | `dnormLog(double x)`             Returns the log-density of the standard normal. |
| `static double` | `dnormLog(double x, double mean, double sd)`             Returns the log-density value of a standard normal. |
| `static DoubleVector` | `dnormLog(double x, DoubleVector mean, double sd)`             Returns the log-density values of a set of normal distributions with different means. |
| `java.lang.String` | `getRevision()`             Returns the revision string. |
| `static double` | `hypot(double a, double b)`             sqrt(a^2 + b^2) without under/overflow. |
| `static double` | `pchisq(double x)`             Returns the cumulative probability of the Chi-squared distribution |
| `static double` | `pchisq(double x, double ncp)`             Returns the cumulative probability of the noncentral Chi-squared distribution. |
| `static DoubleVector` | `pchisq(double x, DoubleVector ncp)`             Returns the cumulative probability of a set of noncentral Chi-squared distributions. |
| `static double` | `pnorm(double x)`             Returns the cumulative probability of the standard normal. |
| `static double` | `pnorm(double x, double mean, double sd)`             Returns the cumulative probability of a normal distribution. |
| `static DoubleVector` | `pnorm(double x, DoubleVector mean, double sd)`             Returns the cumulative probability of a set of normal distributions with different means. |
| `static DoubleVector` | `rchisq(int n, double ncp, java.util.Random random)`             Generates a sample of a Chi-square distribution. |
| `static DoubleVector` | `rnorm(int n, double mean, double sd, java.util.Random random)`             Generates a sample of a normal distribution. |
| `static double` | `square(double x)`             Returns the square of a value |

**Methods inherited from class java.lang.Object**

`equals, getClass, hashCode, notify, notifyAll, toString, wait, wait, wait`

**Field Detail**

### PSI

```
public static final double PSI
```

:   The constant 1 / sqrt(2 pi)

    **See Also:**: Constant Field Values

---


### logPSI

```
public static final double logPSI
```

:   The constant - log( sqrt(2 pi) )

    **See Also:**: Constant Field Values

---


### undefinedDistribution

```
public static final int undefinedDistribution
```

:   Distribution type: undefined

    **See Also:**: Constant Field Values

---


### normalDistribution

```
public static final int normalDistribution
```

:   Distribution type: noraml

    **See Also:**: Constant Field Values

---


### chisqDistribution

```
public static final int chisqDistribution
```

:   Distribution type: chi-squared

    **See Also:**: Constant Field Values


**Constructor Detail**

### Maths

```
public Maths()
```


**Method Detail**

### hypot

```
public static double hypot(double a,
                           double b)
```

:   sqrt(a^2 + b^2) without under/overflow.

---


### square

```
public static double square(double x)
```

:   Returns the square of a value

    :   **Parameters:**: `x` - **Returns:**: the square

---


### pnorm

```
public static double pnorm(double x)
```

:   Returns the cumulative probability of the standard normal.

    :   **Parameters:**: `x` - the quantile

---


### pnorm

```
public static double pnorm(double x,
                           double mean,
                           double sd)
```

:   Returns the cumulative probability of a normal distribution.

    :   **Parameters:**: `x` - the quantile: `mean` - the mean of the normal distribution: `sd` - the standard deviation of the normal distribution.

---


### pnorm

```
public static DoubleVector pnorm(double x,
                                 DoubleVector mean,
                                 double sd)
```

:   Returns the cumulative probability of a set of normal distributions
    with different means.

    :   **Parameters:**: `x` - the vector of quantiles: `mean` - the means of the normal distributions: `sd` - the standard deviation of the normal distribution. **Returns:**: the cumulative probability

---


### dnorm

```
public static double dnorm(double x)
```

:   Returns the density of the standard normal.

    :   **Parameters:**: `x` - the quantile **Returns:**: the density

---


### dnorm

```
public static double dnorm(double x,
                           double mean,
                           double sd)
```

:   Returns the density value of a standard normal.

    :   **Parameters:**: `x` - the quantile: `mean` - the mean of the normal distribution: `sd` - the standard deviation of the normal distribution. **Returns:**: the density

---


### dnorm

```
public static DoubleVector dnorm(double x,
                                 DoubleVector mean,
                                 double sd)
```

:   Returns the density values of a set of normal distributions with
    different means.

    :   **Parameters:**: `x` - the quantile: `mean` - the means of the normal distributions: `sd` - the standard deviation of the normal distribution. **Returns:**: the density

---


### dnormLog

```
public static double dnormLog(double x)
```

:   Returns the log-density of the standard normal.

    :   **Parameters:**: `x` - the quantile **Returns:**: the density

---


### dnormLog

```
public static double dnormLog(double x,
                              double mean,
                              double sd)
```

:   Returns the log-density value of a standard normal.

    :   **Parameters:**: `x` - the quantile: `mean` - the mean of the normal distribution: `sd` - the standard deviation of the normal distribution. **Returns:**: the density

---


### dnormLog

```
public static DoubleVector dnormLog(double x,
                                    DoubleVector mean,
                                    double sd)
```

:   Returns the log-density values of a set of normal distributions with
    different means.

    :   **Parameters:**: `x` - the quantile: `mean` - the means of the normal distributions: `sd` - the standard deviation of the normal distribution. **Returns:**: the density

---


### rnorm

```
public static DoubleVector rnorm(int n,
                                 double mean,
                                 double sd,
                                 java.util.Random random)
```

:   Generates a sample of a normal distribution.

    :   **Parameters:**: `n` - the size of the sample: `mean` - the mean of the normal distribution: `sd` - the standard deviation of the normal distribution.: `random` - the random stream **Returns:**: the sample

---


### pchisq

```
public static double pchisq(double x)
```

:   Returns the cumulative probability of the Chi-squared distribution

    :   **Parameters:**: `x` - the quantile

---


### pchisq

```
public static double pchisq(double x,
                            double ncp)
```

:   Returns the cumulative probability of the noncentral Chi-squared
    distribution.

    :   **Parameters:**: `x` - the quantile: `ncp` - the noncentral parameter

---


### pchisq

```
public static DoubleVector pchisq(double x,
                                  DoubleVector ncp)
```

:   Returns the cumulative probability of a set of noncentral Chi-squared
    distributions.

    :   **Parameters:**: `x` - the quantile: `ncp` - the noncentral parameters

---


### dchisq

```
public static double dchisq(double x)
```

:   Returns the density of the Chi-squared distribution.

    :   **Parameters:**: `x` - the quantile **Returns:**: the density

---


### dchisq

```
public static double dchisq(double x,
                            double ncp)
```

:   Returns the density of the noncentral Chi-squared distribution.

    :   **Parameters:**: `x` - the quantile: `ncp` - the noncentral parameter

---


### dchisq

```
public static DoubleVector dchisq(double x,
                                  DoubleVector ncp)
```

:   Returns the density of the noncentral Chi-squared distribution.

    :   **Parameters:**: `x` - the quantile: `ncp` - the noncentral parameters

---


### dchisqLog

```
public static double dchisqLog(double x)
```

:   Returns the log-density of the noncentral Chi-square distribution.

    :   **Parameters:**: `x` - the quantile **Returns:**: the density

---


### dchisqLog

```
public static double dchisqLog(double x,
                               double ncp)
```

:   Returns the log-density value of a noncentral Chi-square distribution.

    :   **Parameters:**: `x` - the quantile: `ncp` - the noncentral parameter **Returns:**: the density

---


### dchisqLog

```
public static DoubleVector dchisqLog(double x,
                                     DoubleVector ncp)
```

:   Returns the log-density of a set of noncentral Chi-squared
    distributions.

    :   **Parameters:**: `x` - the quantile: `ncp` - the noncentral parameters

---


### rchisq

```
public static DoubleVector rchisq(int n,
                                  double ncp,
                                  java.util.Random random)
```

:   Generates a sample of a Chi-square distribution.

    :   **Parameters:**: `n` - the size of the sample: `ncp` - the noncentral parameter: `random` - the random stream **Returns:**: the sample

---


### getRevision

```
public java.lang.String getRevision()
```

:   Returns the revision string.

    :   **Specified by:**: `getRevision` in interface `RevisionHandler`
    :   **Returns:**: the revision


---


|  |  |  |
| --- | --- | --- |
| **Overview** \| **Package** \| **Class** \| **Tree** \| **Deprecated** \| **Index** \| **Help** \| **Weka's home** | |  |
| **PREV CLASS**   **NEXT CLASS** | **FRAMES**    **NO FRAMES**     **All Classes** |  |
| SUMMARY: NESTED \| FIELD \| CONSTR \| METHOD | DETAIL: FIELD \| CONSTR \| METHOD |  |


---
